# Effect of voluntary exercise upon the metabolic syndrome and gut microbiome composition in mice

**DOI:** 10.14814/phy2.15068

**Published:** 2021-11-09

**Authors:** Timothy M. Moore, Anthony Terrazas, Alexander R. Strumwasser, Amanda J. Lin, Xiaopeng Zhu, Akshay T. S. Anand, Christina Q. Nguyen, Linsey Stiles, Frode Norheim, Jennifer M. Lang, Simon T. Hui, Lorraine P. Turcotte, Zhenqi Zhou

**Affiliations:** ^1^ Division of Cardiology Department of Medicine University of California Los Angeles California USA; ^2^ Department of Human Genetics University of California Los Angeles California USA; ^3^ Division of Endocrinology, Diabetes, and Hypertension University of California Los Angeles California USA; ^4^ Division of Pediatric Endocrinology Department of Pediatrics UCLA Children's Discovery and Innovation Institute Department of Medicine University of California Los Angeles California USA; ^5^ Department of Biological Sciences Dana & David Dornsife College of Letters, Arts, and Sciences University of Southern California Los Angeles California USA; ^6^ Present address: Department of Endocrinology and Metabolism. Zhongshan Hospital Fudan University Shanghai P.R.China; ^7^ Present address: Department of Nutrition Faculty of Medicine Institute of Basic Medical Sciences University of Oslo Oslo Norway

**Keywords:** exercise, LDLR, metabolic syndrome, microbiome, obesity

## Abstract

The metabolic syndrome is a cluster of conditions that increase an individual's risk of developing diseases. Being physically active throughout life is known to reduce the prevalence and onset of some aspects of the metabolic syndrome. Furthermore, previous studies have demonstrated that an individual's gut microbiome composition has a large influence on several aspects of the metabolic syndrome. However, the mechanism(s) by which physical activity may improve metabolic health are not well understood. We sought to determine if endurance exercise is sufficient to prevent or ameliorate the development of the metabolic syndrome and its associated diseases. We also analyzed the impact of physical activity under metabolic syndrome progression upon the gut microbiome composition. Utilizing whole‐body low‐density lipoprotein receptor (LDLR) knockout mice on a “Western Diet,” we show that long‐term exercise acts favorably upon glucose tolerance, adiposity, and liver lipids. Exercise increased mitochondrial abundance in skeletal muscle but did not reduce liver fibrosis, aortic lesion area, or plasma lipids. Lastly, we observed several changes in gut bacteria and their novel associations with metabolic parameters of clinical importance. Altogether, our results indicate that exercise can ameliorate some aspects of the metabolic syndrome progression and alter the gut microbiome composition.

## Graphical abstract

In this study, we sought to determine if endurance exercise is sufficient to prevent or ameliorate the development of metabolic syndrome and its associated diseases and the impact of physical activity under metabolic syndrome progression upon the gut microbiome. Utilizing the whole‐body low‐density lipoprotein receptor (LDLR) knockout mice on a “Western Diet,” we show that long‐term exercise acts favorably upon glucose tolerance, adiposity, and liver lipids. Our results indicate that exercise can ameliorate some aspects of the metabolic syndrome progression and alter the gut microbiome.

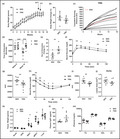

## 1 INTRODUCTION

The metabolic syndrome, including its sequelae of heart disease, atherosclerosis, insulin resistance, non‐alcoholic fatty liver disease (NAFLD), and others, is a burgeoning health crisis that affects an estimated one‐third of adults and an increasing number of children in developed countries (Cohen et al., 2011). Although combination and single drug therapies are under evaluation, no pharmacological treatment is approved to treat all, a majority, or even a few aspects of the metabolic syndrome (Rask Larsen et al., 2018). The current mainstay therapy to combat the metabolic syndrome is via lifestyle modifications, primarily through diet alterations and increased physical activity, in an attempt to lower whole‐body adiposity, reduce chronic hyperglycemia, prevent blood vessel plaque formation, and dyslipidemia (Fiuza‐Luces et al., 2018). Clinical trials have demonstrated the ability of physical activity to reduce hepatic lipid content and improve insulin sensitivity in humans (Winn et al., 2018). However, not all mechanism(s) by which exercise improves whole‐body metabolism especially in the context of progressing or preexisting metabolic syndrome are known (Balducci et al., 2015; El‐Agroudy et al., 2019; Hallsworth et al., 2011; Pugh et al., 2013, 2014). Hence, understanding the molecular mechanisms underlying the metabolic syndrome and its progression in the context of an exercise intervention is urgent for the development of effective therapies and reducing the overall burden upon societies, health care systems, families, and individuals.

Recently, a host's gut microbiome has received increasing attention and found to influence several aspects of the metabolic syndrome and its associated diseases (Chen & Devaraj, 2018; Dabke et al., 2019). The gut microbiome is also beginning to be studied following exercise interventions with recent reports suggesting an intimate link between the gut microbiome and the benefits of physical activity (Hawley, 2020; Liu et al., 2020; Mach & Fuster‐Botella, 2017; Ortiz‐Alvarez et al., 2020; Scheiman et al., 2019). Such an area of research is exciting given the potential impact upon public and individual health (Lai et al., 2018; Pedersini et al., 2020). While the mechanisms under non‐disease or athletic conditions are currently being elucidated, what has received little research attention is whether being physically active can preserve or promote a diverse gut microbiome in the context of diet‐induced metabolic syndrome and whether this impacts organismal metabolic health (Allen et al., 2018; Carbajo‐Pescador et al., 2019; Denou et al., 2016; Rettedal et al., 2020).

We sought to determine if endurance exercise is sufficient to prevent or ameliorate the development of the metabolic syndrome and its associated diseases. We also pursued the impact of physical activity under metabolic syndrome progression upon the gut microbiome. To address our research question, we subjected whole‐body low‐density lipoprotein receptor knockout mice (LDLR^−/−^) on a “Western diet” (rich in saturated fat, cholesterol, and refined carbohydrates) to a voluntary endurance exercise protocol. We hypothesized that endurance exercise would protect mice from the metabolic syndrome and would modify the gut microbiome. We found that long‐term exercise training preserved cardiometabolic fitness and glucose homeostasis while also reducing adiposity and liver lipid accumulation. Several gut bacteria (operational taxonomic units) were significantly altered and strongly associated with markers of clinical importance. We conclude that exercise has an effect upon the gut microbiome during the progression and onset of the metabolic syndrome and continue to affirm the positive effects of exercise upon organismal metabolic health.

## 2 METHODS

### 2.1 Animal studies

All animal studies were conducted at the University of California, Los Angeles and were approved by the affiliated Institutional Animal Care and Use Committee (ARC‐2007‐051). All animal care, maintenance, surgery, and euthanasia were conducted in accordance with UCLA's Institutional Animal Care and Use Committee and the National Institutes of Health.

Male, whole‐body low‐density lipoprotein receptor knockout mice (LDLR^−/−^) were obtained from Jackson Labs (002207) and maintained on a strict 12‐h light–dark cycle, standard vivarium housing temperatures (18–23℃), and relative humidity (40%–60%). All mice were on an ad libitum water and normal rodent chow diet until approximately 4 months of age. Animals were randomly divided into two groups (SED = sedentary or no exercise and TRN = voluntary exercise training) and placed on an ad libitum water and “Western Diet” (% by weight: 33% kcal fat, 18% kcal protein, 48% kcal carbohydrate, 1% cholesterol; Research Diets D10042101) for 16 weeks (*N* = 10/group). This model was chosen as it has been shown that genetic and dietary manipulations are effective at inducing NAFLD in the mouse (Oligschlaeger & Shiri‐Sverdlov, 2020; Wouters et al., 2005, 2008) and the development of the metabolic syndrome (Grundy et al., 2004). Animals in TRN were given unlimited access to an in cage running wheel. Wheel revolutions were monitored using VitalView® Activity Software (Starr Life Sciences Corp). TRN animals were singly housed for the duration of the experiment. SED animals were housed in original cages at 1–4 per cages. Thirty hours prior to euthanasia, in cage running wheels were locked. All animals were fasted for 6 h prior to euthanasia. Animals were sacrificed with a lethal dose of isoflurane followed by cervical dislocation. Tissues (quadriceps, gastrocnemius–plantaris–soleus, inguinal white adipose, gonadal white adipose, and liver) were removed, rinsed in 0.9% saline, blotted dry, weighed, frozen in liquid nitrogen, and stored at −80℃ until use. Cecum, samples used for the conduction of 16S analysis, was not rinsed in saline. A portion from the large lobe of the liver and aorta was fixed for histological analysis before being frozen in liquid nitrogen.

### 2.2 Exercise capacity test

The exercise capacity test was performed as described previously (Moore et al., 2019). Exercise capacity tests were performed prior to initiation of the experiment and 3 days prior to euthanasia. Mice were removed from home cages and randomly paired into clean cages without food approximately 3 h prior to the exercise capacity test. Testing personnel were blinded to mouse groups. Mice were acclimated to the running treadmill on three separate occasions prior to performing each test. Following a brief warm up, all mice completed a run to exhaustion test starting at 10 m/min (fixed 5° incline) with speed increased by 3 m/min every 3 min. The test was terminated when mice were no longer able to perform the test as indicated by >10 consecutive seconds upon the resting platform despite gentle encouragement using a large tongue depressor.

### 2.3 Glucose and insulin tolerance tests

Glucose and insulin tolerance tests were performed at weeks 14 and 15 of the experimental protocol, respectively. Animals were fasted overnight (approximately 16 h) prior to the glucose tolerance test and 8 h prior to the insulin tolerance test. A shorter fast was used for the insulin tolerance test to ensure mice did not reach life‐threatening hypoglycemia. In cage, running wheels were locked the night before each test to avoid the effects of the most recent exercise bout which would have occurred ~30 h prior. Tests were performed as described previously (Ribas et al., 2016). For the glucose tolerance test, an intraperitoneal dextrose (1 g/kg) injection dissolved in saline was administered. For the insulin tolerance test, an injection of intraperitoneal insulin (0.7 U/kg) dissolved in saline was administered. Glucose was measured from whole blood at 0, 15, 30, 45, 60, 90, and 120 min post‐injection.

### 2.4 Lesion area

Aortic lesions were determined as described previously (Bennett et al., 2015). Briefly, excised aortas were flushed with ice‐cold PBS, embedded in chilled OCT, frozen on dry ice, and stored at −80℃ until use. Embedded aortas were sectioned into 10 µm slices and stained with Oil Red O. Lesion area was quantified in every third section throughout the aorta.

### 2.5 Plasma analysis

Whole blood obtained from retro‐orbital bleeding was placed into EDTA‐coated tubes and spun at 3000 G for 5 min. The resulting plasma supernatant was collected, frozen in liquid nitrogen, and stored at −80℃ until use. Plasma triglycerides and glucose were measured as described previously (Castellani et al., 2008).

### 2.6 Liver histology

Livers sections were obtained, embedded, and sectioned as described previously (Hui et al., 2018). Briefly, liver tissues were fixed in phosphate‐buffered 10% formalin and embedded in paraffin wax. Sections were cut and stained with hematoxylin & eosin (H&E) and Masson's trichrome. Histology and fibrosis scores were applied to assess the severity of liver fibrosis (score 0: none; 1: perisinusoidal or periportal fibrosis; 2: perisinusoidal and portal/periportal fibrosis; 3: bridging fibrosis; and 4: cirrhosis) (Kleiner et al., 2005). Scores were given based upon the staining of the whole slides. Scorers were blinded to mouse group.

### 2.7 Tissue lipids

Lipids were isolated and analyzed from either liver or gonadal white adipose tissue as described previously (Hui et al., 2015). Briefly, 50–100 mg of tissue was homogenized in methanol after which chloroform was added making a final solution of 2:1 methanol:chloroform. Samples were rotated overnight at 4℃ after which they were filtered through sharkskin filter paper. A 0.043% magnesium chloride solution was added, centrifuged at 650×g, and the remaining solution was dried using nitrogen gas. A solution of 1.8% Triton X‐100 dissolved in water was then added after which the following lipids were measured: triglyceride, total cholesterol, high‐density lipoprotein (HDL), unesterified cholesterol, cholesterol ester, and phosphatidylcholine.

### 2.8 DNA and RNA analysis

DNA or RNA was isolated from frozen tissue using the respective DNA or RNA isolation kit following the manufacturer's instructions (for DNA: DNeasy Blood & Tissue Kit (69504), for RNA: RNeasy Kit (74104), Qiagen). Isolated DNA or RNA was tested for concentration and purity using a NanoDrop Spectrophotometer. Mitochondrial DNA content was determined by the ratio of *mtCO2* (mitochondrial‐derived gene) to *18S* (nuclear DNA‐derived gene). Isolated RNA was converted to cDNA (iScript Reverse Transcription Supermix for RT‐qPCR (1708840), Bio‐Rad) before qPCR was performed for specific genes (*Acc1*, *Acox1*, *Acta2*, *Atg3*, *Atg5*, *Atgl*, *Col1a1*, *Cyp8b1*, *Fasn*, *Hsl*, *Il18*, *Il1b*, *Il6*, *Insig2*, *Lxra*, *Map1lcb3*, *Mcp1*, *mtCo1*, *mtCo3*, *mtNd4*, *Park2*, *Park7*, *Pgc1a*, *Pink1*, *Polrmt*, *Ppara*, *Qsox1*, *Scd1*, *Spstm1*, *Tnfa*, or *Ucp1*). See Table S1 for a list of the primers used and corresponding sequences. All genes were normalized to *18S* or *Ppia* where indicated and expressed relative to SED (sedentary or unexercised) group average.

### 2.9 Mitochondrial respiration

Mitochondrial respiration was measured in frozen biological samples as described previously (Acin‐Perez et al., 2020). Frozen tissues were thawed on ice and homogenized in MAS (70 mM sucrose, 220 mM mannitol, 5 mM KH2PO4, 5 mM MgCl2, 1 mM EGTA, 2 mM HEPES, pH 7.4). The samples were mechanically homogenized for 60 strokes in a Teflon‐glass dounce homogenizer. All homogenates were centrifuged at 1000×g for 10 min at 4℃ and then the supernatant was collected. Protein concentration was determined by BCA (Thermo Scientific). Homogenates were loaded into Seahorse XF96 microplate in 20 μl of MAS at 6 µg/well. The loaded plate was centrifuged at 2400×g for 10 min at 4℃ (no brake) and an additional 130 μl of MAS supplemented with 100 µg/ml cytochrome c was added to each well. Substrate injection was as follows: Port A: NADH (1 mM) or succinate + rotenone (5 mM + 2 μM); Port B: rotenone + antimycin A (2 μM + 2 μM); Port C: N,N,N',N'‐tetramethyl‐p‐phenylenediamine (TMPD) + ascorbic acid (0.5 mM + 1 mM); and Port D: azide (50 mM). These conditions allow for the determination of the maximal respiratory capacity of mitochondria through Complex I, Complex II, and Complex IV.

### 2.10 Immunoblotting

Frozen tissues were pulverized at the temperature of liquid nitrogen and a homogenous portion of the entire tissue was used for immunoblotting. Proteins were normalized to GAPDH (glyceraldehyde 3‐phosphate dehydrogenase) or HSP90 (heat shock protein 90) where indicated and expressed relative to SED (sedentary or unexercised) group average.

### 2.11 Microbiome analysis

Profiling of the microbiome was completed as described previously using mouse cecum (Org et al., 2015). DNA was isolated from approximately 75 mg of cecum using the DNeasy PowerSoil Kit (Qiagen) following the manufacturer's instructions. Isolated DNA was checked for concentration using a Nanodrop spectrophotometer. 16S rRNA V4 region primers were used during PCR amplification (Caporaso et al., 2012). The primer sequences used without linker, pad, barcode, or adapter are Forward: GTGCCAGCMGCCGCGGTAA and Reverse: GGACTACHVGGGTWTCTAAT. PCR products were verified, quantified, and pooled before a final clean up using UltraClean PCR Clean‐Up Kit before subsequent sequencing on an Illumina HiSeq 3000. All raw fastq files have been deposited in NCBI SRA under PRJNA759241.

Fastq files were trimmed for primers and analyzed using the DADA2 pipeline (Callahan et al., 2016). Taxonomy was assigned using Silva v138 (Quast et al., 2013). Raw ASV counts were analyzed for differential expression using the DESeq2 (Version 1.28.1) R package (Love et al., 2014). ASVs were considered significant when FDR (false discovery rate) <0.1. Alpha diversity was calculated using the “estimate_richness” function within the phyloseq (Version 1.32.0) R package (McMurdie & Holmes, 2013).

### 2.12 Statistics

Values are presented as means ± SEM and expressed relative to SED (sedentary or unexercised) group average unless otherwise stated. Correlations were computed using the biweight midcorrelation (bicor) function within the weighted gene correlation network analysis (WGNCA) R Package (Version 1.66) (Langfelder & Horvath, 2012). Statistical significance was computed via a two‐tailed independent *t*‐test and established a priori at *p* < 0.05 unless otherwise stated. Graphs were made using GraphPad Prism 8.4.2 (GraphPad Software) or R/R Studio (Version 4.0.0/ Version 1.3.959). *p < 0.05, **p < 0.01, and ***p < 0.001.

## 3 RESULTS

### 3.1 Exercise training maintains exercise capacity and impedes progression of the metabolic syndrome

**FIGURE 1:**
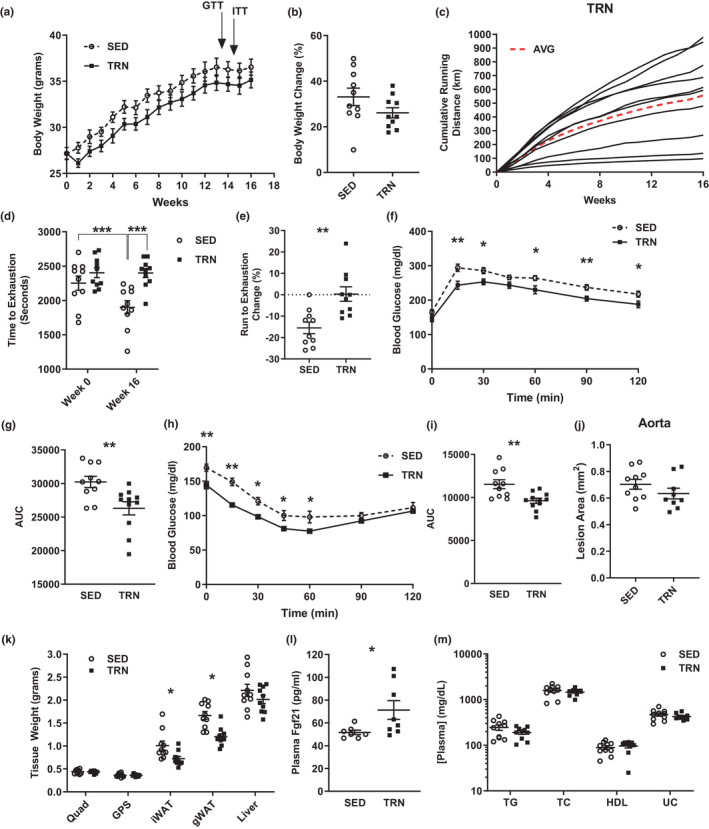
(a) Weekly body weight. (b) Change in body weight from week 0 to week 16. (c) Cumulative running distance per week in km. Red dashed line represents overall cumulative weekly average. (d) Time to exhaustion during exercise test. (e) Change in run to exhaustion expressed as a percent of each animal's week 0 time. (f) Blood glucose levels during the glucose tolerance test at week 14. (g) Area under the curve from glucose tolerance test. (h) Blood glucose levels during insulin tolerance test at week 15. (i) Area under the curve during the insulin tolerance test at week 15. (j) Aorta lesion area. (K) Wet weight of each tissue at the time of sacrifice. (l) Fgf21 levels in plasma at the time of sacrifice. (m) Plasma metabolite concentration at the time of sacrifice. Groups represented as mean ± SEM. *N* = 7–10/group. *p < 0.05, **p < 0.01, and ***p < 0.001

After 16 weeks, exercise‐trained mice (TRN) displayed no significant difference in body weight compared to sedentary mice (SED, *p *> 0.05, Figure 1a,b). On average, TRN mice ran approximately 500 km over the duration of the experiment, or ~4.5 km/day (Figure 1c). Prior to running wheel access (week 0), there was no difference in exercise capacity between groups (*p *> 0.05, Figure 1d). After the 16‐week experimental protocol, exercise capacity was significantly higher in TRN versus SED mice (*p *< 0.001, Figure 1d,e). Glucose and insulin tolerance tests (GTT and ITT, respectively) showed an increased ability of TRN mice to clear glucose and maintain euglycemia (*p *< 0.05, Figure 1f–i). Aortic lesion area was not significantly different between groups (*p *> 0.05, Figure 1j). TRN mice showed lower inguinal white adipose tissue (iWAT) and gonadal white adipose tissue (gWAT) masses (*p *< 0.05, Figure 1k). Additionally, plasma levels of Fgf21 (fibroblast growth factor 21), a recently identified myokine with implications in fatty liver disease (Tezze et al., 2019), were elevated in TRN mice (*p *< 0.05, Figure 1l). There were no significant differences observed for plasma lipids (*p *> 0.05, Figure 1m).

### 3.2 Exercise training reduces lipid levels within the liver during metabolic syndrome progression

**FIGURE 2:**
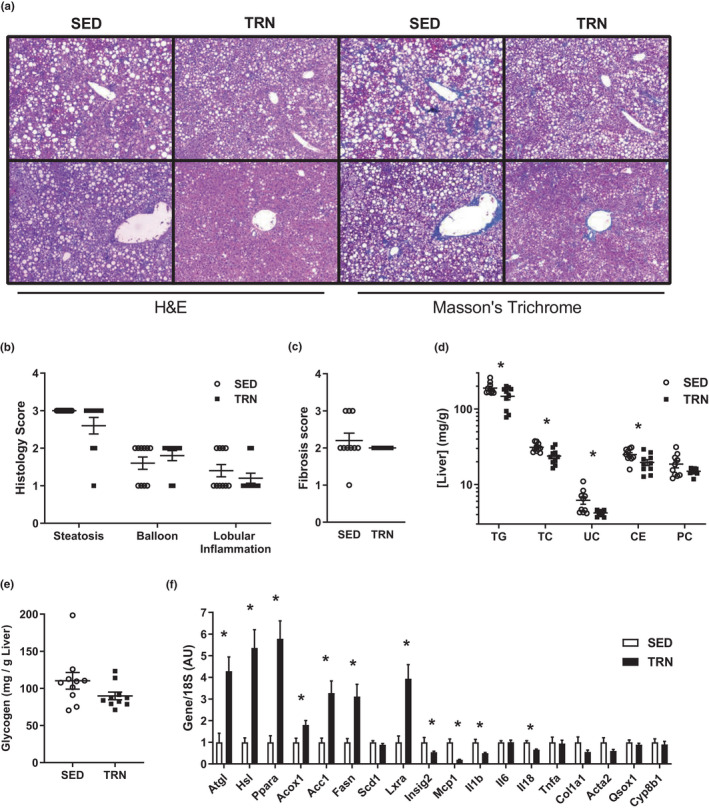
(a) Liver H&E (left two columns) and Masson's Trichrome (right two columns) images. (b, c) Histology and fibrosis score from panel A. (d, e) Lipid or glycogen concentrations within liver. (f) Gene expression within liver normalized to the housekeeping gene 18S and expressed relative to SED avg. Groups represented as mean ± SEM. *N* = 10/group. *p < 0.05

No significant differences were observed in histology and fibrosis scores between SED and TRN mice after the experimental protocol (*p *> 0.05, Figure 2b,c). Nevertheless, decreases in triglyceride (TG), total cholesterol (TC), unesterified cholesterol (UC), and cholesterol ester (CE) in the liver were observed in TRN mice (*p *< 0.05, Figure 2d). Glycogen levels were similar between groups (*p *> 0.05, Figure 2e). The expression of genes related to fatty acids (*Atgl*, *Hsl*, *Pparα*, *Acox1*, *Fasn*, *Scd1*, *Lxrα*, *Insig2*, and *Cyp8b1*), inflammation (*Mcp1*, *Il1β*, *and Il6*, *Il18*, and *Tnfα*), and fibrosis (*Col1a1*, *Acta2*, and *Qsox1*) revealed an increase in those related to fatty acid metabolism and a decrease in inflammatory genes between TRN and SED mice (*p *< 0.05, Figure 2f).

### 3.3 Exercise training does not alter liver mitochondrial function during metabolic syndrome progression

**FIGURE 3:**
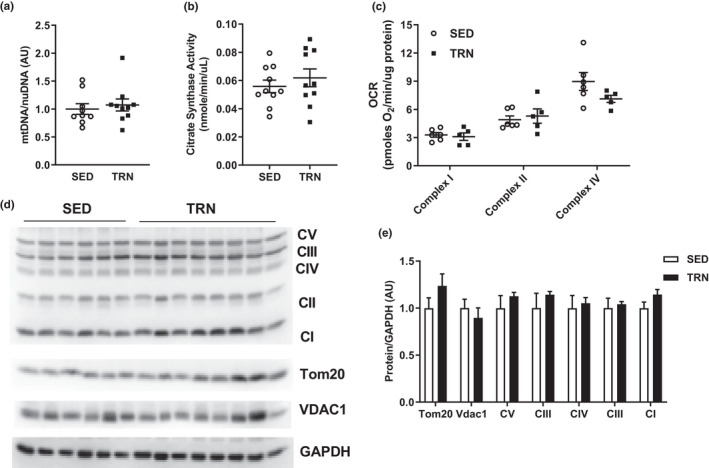
(a) Mitochondrial DNA to nuclear DNA ratio within liver expressed relative to SED average. (b) Citrate synthase activity from frozen liver expressed as nanomoles/minute/microgram of liver. (c) Oxygen consumption rate from frozen liver expressed as picomoles of oxygen/minute/microgram of protein. (d, e) Immunoblot with resulting densitometry within liver normalized to the housekeeping protein GAPDH and expressed relative to SED group average. Groups represented as mean ± SEM. *N* = 5–10/group

We next sought to determine the impact of physical activity upon mitochondrial function within the liver under the metabolic syndrome condition. Mitochondrial DNA copy number, citrate synthase activity, and oxygen consumption were not significantly different between groups after 16 weeks of voluntary physical activity (*p *> 0.05, Figure 3a–c). Furthermore, immunoblotting for mitochondrial proteins revealed no difference between groups (*p *> 0.05, Figure 3d,e).

### 3.4 Exercise training increases skeletal muscle mitochondrial abundance during metabolic syndrome progression

**FIGURE 4:**
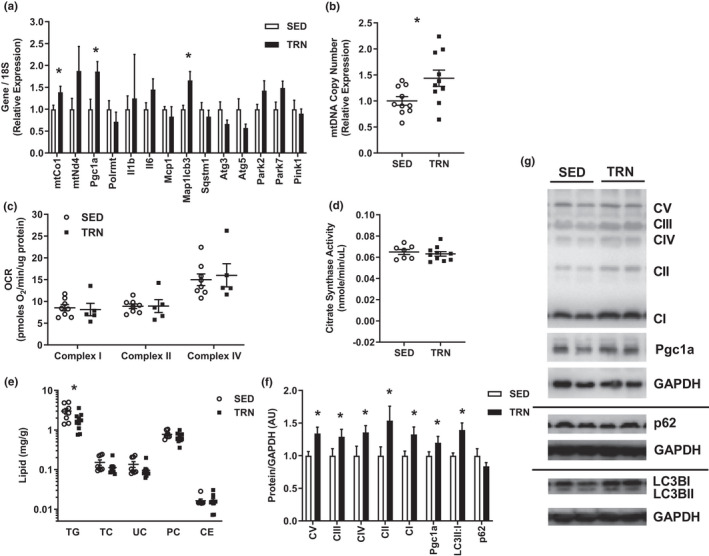
(a) Gene expression within quadriceps muscle normalized to the housekeeping gene 18S and expressed relative to SED average. (b) Mitochondrial DNA to nuclear DNA ratio within quadriceps muscle expressed relative to SED average. (c) Oxygen consumption rate from frozen quadriceps muscle expressed as picomoles of oxygen/minute/microgram of protein. (d) Citrate synthase activity from frozen quadriceps muscle expressed as nanomoles/minute/microgram of muscle. (e) Lipid concentrations within quadriceps muscle. (f, g) Immunoblot with resulting densitometry within quadriceps muscle normalized to the housekeeping protein GAPDH and expressed relative to SED group average (Showing *N* = 2/group). Groups represented as mean ±SEM. *N* = 5–10/group. *p < 0.05

We further assessed mitochondrial function within skeletal muscle. Gene expression within the quadriceps muscle revealed an increase in genes related to mitochondria (*mtCo1*) and mitochondrial biogenesis (*Pgc1a*) between TRN and SED mice (*p *< 0.05, Figure 4a). We also observed an increase in mitochondrial DNA copy number in the TRN versus SED groups (*p *< 0.05, Figure 4b). Nevertheless, we did not observe a difference in oxygen consumption or citrate synthase activity between groups (*p *> .05, Figure 4c,d), a result that could be due to the excess intracellular lipids feeding back to reduce mitochondrial ATP production. A decrease in triglyceride levels within muscle was also observed in TRN mice (*p *< *0*.*05*, Figure 4e). Immunoblotting revealed an increase in mitochondrial proteins in TRN relative to SED mice (*p *< 0.05, Figure 4f,g).

### 3.5 Exercise training induces Ucp1 in white adipose tissue during metabolic syndrome progression

**FIGURE 5:**
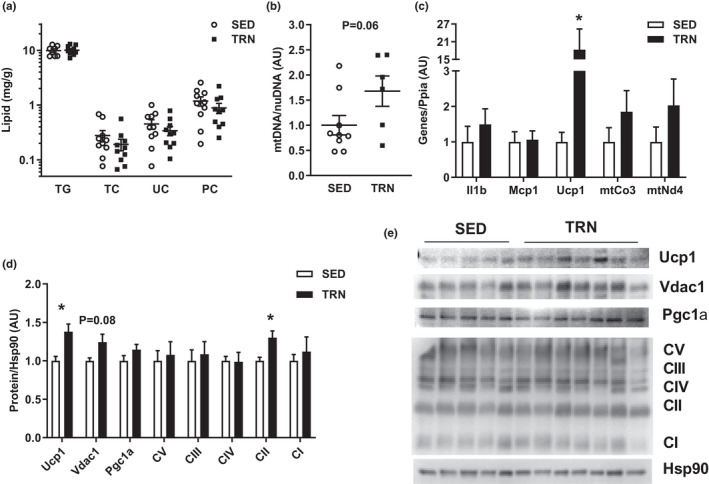
(a) Lipid concentrations within iWAT. (b) Mitochondrial DNA to nuclear DNA ratio within iWAT expressed relative to SED average. (c) Gene expression within iWAT normalized to the housekeeping gene Ppia and expressed relative to SED average. (d, e) Immunoblot with resulting densitometry within iWAT normalized to the housekeeping protein Hsp90 and expressed relative to SED group average (Showing *N* = 2/group). Groups represented as mean ± SEM. *N* = 5–10/group. *p < 0.05

Lipid species concentrations within inguinal white adipose tissue (iWAT) were unchanged between groups (*p *> 0.05, Figure 5a). TRN mice exhibited a non‐significant increase in mitochondrial DNA copy number (*p *= 0.06, Figure 5b). TRN mice further displayed an increase in uncoupling protein 1 (Ucp1) at the gene and protein level when compared to SED mice (*p *< 0.05, Figure 5c–e).

### 3.6 Exercise training impacts specific gut microbes during the progression of the metabolic syndrome

**FIGURE 6:**
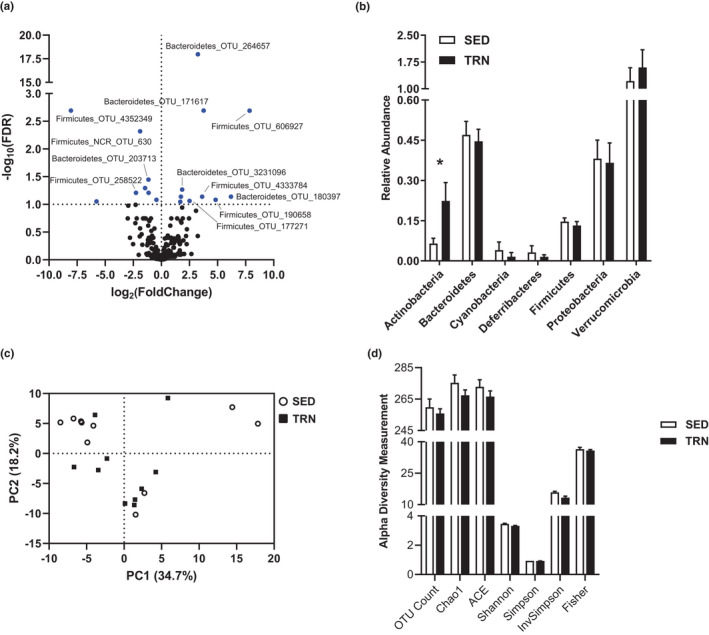
(a) Volcano plot of OTUs identified within the cecum. Significant OTUs are colored blue. (b) Phylum level summary of OTUs. (c) Unbiased principal component analysis. (d) Select measures of alpha diversity relative to SED group average. Groups represented as mean ± SEM. *N* = 10/group. *p < 0.05, **p < 0.01

We next sought to determine whether physical activity has an effect upon the gut microbiome during the progression of the metabolic syndrome by examining mouse cecum. Eighteen significantly different operational taxonomic units (OTUs) were identified between the groups (FDR < 0.1, Figure 6a). These OTUs were highlighted by Bacteroidetes OTU 264657, which increased nearly 10‐fold (FDR = 1.09 × 10^−18^). Although there were changes in individual OTUs, only the Actinobacteria phylum was significantly affected by exercise training (*p *< 0.05, Figure 6b). Principal component analysis showed that the gut microbiota composition between SED and TRN mice was not statistically different (*p *> 0.05, Figure 6c). Several common measures of alpha diversity revealed no differences between TRN and SED mice (*p *> 0.05, Figure 6d).

### 3.7 Gut microbes are associated with exercise capacity

**FIGURE 7:**
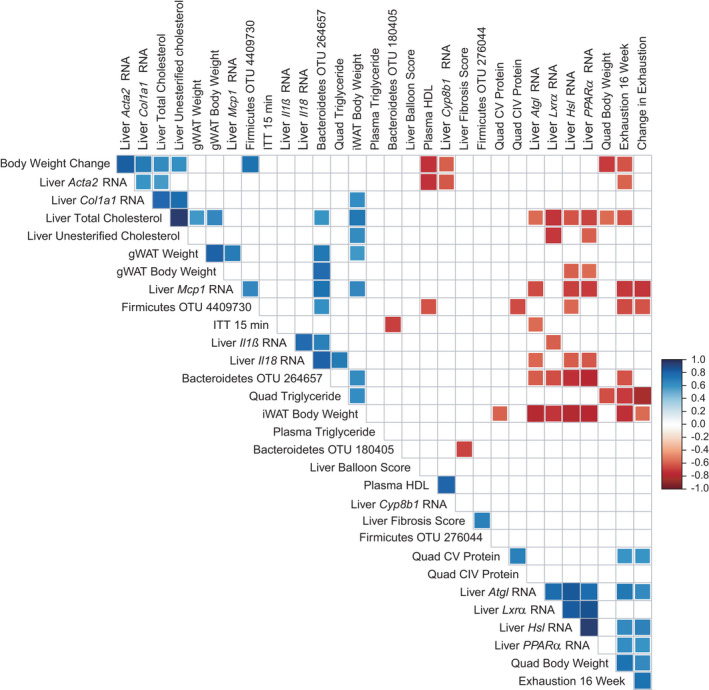
Correlation matrix. Only factors with one or more significant associations are shown (*p *< 0.01). Red = negative association and blue = positive association

We then combined groups (SED & TRN) and correlated all traits displaying only significant correlations via a heatmap (Figure 7) to uncover novel relationships. A significance threshold of *p *< 0.01 was used to better account for the large number of correlations performed. We observed several expected correlations such as fat mass with exercise performance. We also observed correlations between gut microbes and exercise capacity. Furthermore, we observed relationships between liver gene expression with gut microbes. Bacteroidetes OTU 264657, the most significantly changed OTU between groups, displayed multiple significant correlations with fat mass, liver cholesterol, liver fatty acid and inflammation‐associated genes, and exercise performance.

## 4 DISCUSSION

We sought to elucidate whether exercise is sufficient to prevent or ameliorate the progression or onset of the metabolic syndrome and NAFLD. By utilizing a severe model of metabolic syndrome progression (genetic and dietary intervention), we found that endurance exercise was able to prevent some aspects of the metabolic syndrome that developed in sedentary mice over the 16‐week period. Notably, exercise maintained exercise capacity and enhanced glucose tolerance while reducing adiposity, liver lipids, and glucose level during insulin tolerance testing. Our examination of the gut microbiome also revealed several bacterial species to be significantly impacted with exercise training.

The metabolic syndrome is a cluster of conditions that include insulin resistance, obesity, atherosclerosis, and NAFLD (Kennedy et al., 2010). Our main goal was to determine if exercise could prevent the onset of the metabolic syndrome. In alignment with previous research, we found that chronic physical activity could improve some aspects of metabolic health even under the context of a severe metabolic syndrome model associated with high adiposity and elevated circulating glucose levels (Joseph et al., 2019; Paley & Johnson, 2018). However, sustained physical activity did not reduce aortic lesion area, a common precursor to an ischemic event. Large epidemiological studies have shown a strong connection between physical activity and coronary artery disease (Winzer et al., 2018). Despite our results, studies examining physical activity under the context of prior atherosclerosis suggest a small reduction in lesion size, though these studies are typically coupled with dietary and other life style components (Ornish et al., 1990; Ramachandran et al., 2005). Furthermore, there is mounting evidence suggesting athletes (meaning those who participate in moderate to vigorous exercise for years or decades) have a higher prevalence of coronary artery calcification (Aengevaeren et al., 2020). While such findings are controversial, they could be applicable to our model given the chronic physical activity of mice (~4.5 km/day for 16 weeks), a substantial period of the mouse life span, consequently masking any changes in aortic lesion area that we observed.

Focusing upon NAFLD, in our study, exercise exerted no significant effect on liver fibrosis as assessed by histology and pathology score. However, a significant reduction in lipid levels and increases in lipid metabolism gene expression were observed. The reduction in lipid levels suggest that exercise may be able to prevent aspects of NAFLD and thus partially preserve liver function during disease progression. Previous research in humans and mice have found similar results regarding liver lipids (Houghton et al., 2017; van der Windt et al., 2018). Nevertheless, the lack of liver collagen reduction suggests exercise is not able to prevent the onset of liver fibrosis and that diet may have a larger effect on NAFLD than exercise, a hypothesis supported by previous research (Kenneally et al., 2017; Ok et al., 2018; Romero‐Gómez et al., 2017).

We also pursued the impact of physical activity under metabolic syndrome progression upon the gut microbiome. Previous research has shown that differences in gut microbiota composition due to diet have an effect on lipid accumulation and onset of NAFLD (Le Roy et al., 2013). In our study, exercise‐trained mice displayed changes within the microbiome. However, there were not significant community wide changes compared to sedentary mice. Additional research has shown minimal changes in the gut microbiome following exercise, although not under the context of severe metabolic syndrome (Kern et al., 2020; Taniguchi et al., 2018). The data presented herein substantiate the notion that dietary factors such as those associated with a “Western diet” have a significant impact on the gut microbiome (Cignarella et al., 2018; Quercia et al., 2017). Nevertheless, given that animals are consuming identical diets, this raises the possibility that a secreted factor(s) is able to traverse the intestinal barrier and impact specific bacterial species, a hypothesis under current investigation (Barger et al., 2020; Dalton et al., 2019; de Sire et al., 2018).

Our correlation analyses uncovered several known and novel associations. For example, we observed known negative associations between adiposity and exercise capacity parameters. We also observed novel significant relationships between exercise capacity parameters and liver phenotypes like *Acta2* gene expression, total cholesterol, *Mcp1* gene expression, and fatty acid metabolism gene expression. These data suggest connections between the liver and exercise capacity further strengthening the concept that exercise promotes liver health. In addition, several bacterial OTUs were strongly associated with exercise capacity and other clinical parameters strengthening the relationship between physical activity and the gut microbiome. Additional research should focus on these OTUs and how they impact organismal physiology.

Although our results indicate that exercise is an effective therapeutic that mitigates the progression of some aspects of the metabolic dysfunction associated with the consumption of a Western diet, our study was restricted to male mice, maintaining the possibility that the results should not be generalized to female mice. We also cannot rule out the effect of chronic exercise training upon feeding as it has been shown that wheel running tends to reduce food intake (Cordeira & Monahan, 2019) or the single housing of exercising mice impacting our phenotypes (Nagy et al., 2002). Lastly, it is possible that the effects of exercise are, at least in part, regulated by LDLR. Thus, this genetic model could have a reduced exercise response. Despite these potential limitations, research has shown that men are at a higher risk to develop NAFLD compared to women (Ballestri et al., 2017), and that environmental factors and lifestyle habits seem to have a more significant impact than genetics (Ahmed et al., 2019).

Altogether, our results suggest that exercise is an effective therapeutic strategy that should be implemented to prevent and alleviate aspects of the metabolic syndrome. However, our results also support that exercise is not able to improve or prevent the onset of all aspects of the metabolic syndrome and the development of NAFLD. In addition, while exercise may have a minimal effect on gut microbiota composition, novel associations between bacterial OTUs and metabolic and performance traits were observed. Further research should examine how the exercise‐trained gut microbiome may reduce the development of the metabolic syndrome and its complications.

## CONFLICT OF INTEREST

The authors declare that they have no competing interests.

## AUTHOR CONTRIBUTIONS

The experimental conception and design were performed by TMM, FN, and ZZ. The animal experiments, sample collection, and subsequent experimental analysis were conducted by TMM, ARS, AJL, ATSA, CQN, LS, FN, JML, ZIK, LPT, STH, and ZZ. The manuscript was originally drafted by TMM, AT, and ZZ. All authors contributed to the final drafting of the manuscript.

## Supporting information



Table S1
